# Supplementary material for: Comparability of modern contraceptive use estimates between a face-to-face survey and a cellphone survey among women in Burkina Faso
Source: PLoS One. 2020 May 13;15(5):e0231819. doi: 10.1371/journal.pone.0231819 (PMC7219703; doi:10.1371/journal.pone.0231819)
Supplement: S1 Data — (DOCX) [file pone.0231819.s001.docx]

| **Burkina Faso Random Digit Dial Survey** |
| --- |

| **NO** | **QUESTIONS AND FILTERS** | **CODING CATEGORIES** | **SKIP** |
| --- | --- | --- | --- |
| **Section 0 – Identification** | | | |
| 1 | Please specify your interviewer number (1-20)  *(not asked to respondent)* | \| Number \|  \| \| --- \| --- \| |  |
| 2 | Hello! My name is (........................), What language would you like to speak? We are currently conducting a health study. We dial the numbers randomly and we had the chance to call your number. | Same language as the interviewer 1    Language different from the 7 options available (*Thank you for your time and have a good evening!*) 2  Language other than interviewer's language *(Add to group: Call back in [language])* 3  Call back later (*same language as interviewer)* 4  Hang up the phone 5  Verbal refusal (*Thank you for your time and have a good evening!*) 6 | 1 go to Q3  2 go to M4a  3 go to Q31  4 go to Q29  5 go to Q31  6 go to Q31 |
| 3 | Are you a man or a woman? | Woman 1  Man (*Thank you, sir, for your time to listen to me, other than that this is a study on women's health and it is addressed only to women. Thank you and have a good evening!)* 2  Call back later (*Thank you for your time, we will call you back later) (Add to group: call back x)* 3    Verbal refusal (*Thank you for your time and have a good evening!*) 4  Hang up 5 | 1 go to Q4  2 go to Q31  3 go to Q29  4 go to M4c  5 go to M4c |
| 4 | Your answers to all my questions are strictly confidential and anonymous. How old are you? | \| Number \|  \| \| --- \| --- \|   Don't know 99  Call back later 90  Refusal Verbal 88  Hang up 77 | <15 or >49 go to M4a  15-49 go to Q5  99 go to M4c  77 or 88 go to M4c  90 go to Q29 |
| M4a | Thank you Madam for your time to respond, but you are not eligible to participate in this study. Thank you and have a good evening. |  | Go to Q31 |
| M4b | Thank you for your time, we will call you back later. (Add to group: recall x) |  | Go to Q31 |
| M4c | Thank you for your time and have a good evening! |  | Go to Q31 |
| Q5 | Do you live in a city or village?  ***Probe***: For example, a city like Ouaga, Bobo, Koudougou, Fada or a rural village? | Urban 0  Rural 1  Do not know 2  Call back late 3  Verbal refusal 4  Hang up 5 | 1,2 go to Q6 4 go to Q29 3, 5, 6 go to Q31 |
| **Informed Consent** | | | |
| Q6 | You are eligible to participate in this study.  I am calling you on behalf of the *l'Institut Supérieur des Sciences de la Population* regarding a study on women's health. We call you to invite you to complete a survey. We will use your answers, as well as those of other women, to improve women's health. You are not required to complete this survey, but if you agree to participate, your responses will be confidential. This investigation takes 10 minutes and you can stop it at any time. I will send you the equivalent of 500 FCFA of telephone credit to compensate you for the time you will devote to this study. Do you agree to participate? | Yes 1  No 0 | 1 go to Q9  0 go to Q7 |
| Q7 | What is the respondent's hesitation? | Too busy, specific reason 1  Too busy for no reason 2  Not confident in the study 3  Other 4 |  |
| Q8 | What is the outcome of your consent discussion? | Consented (continue survey) 1  Not consented (end survey/ do not call back) 2  Call back 3 | 1 go to Q9  2 go to Q31 3 go to Q29 |

|  |  |  |  |
| --- | --- | --- | --- |

| **Section 1 – Respondent’s Background, Marital Status, HH characteristics** | | | | |
| --- | --- | --- | --- | --- |
| Q10 | | Have you ever been to school? | Yes 1  No 2  No response 3 |  |
| Q11 | | What is the highest level of school you attended? | Never Attended 1  Primary 2  Secondary (1^st^ cycle) 3  Secondary (2^nd^ cycle) 4  Tertiary 5  No response -99 |  |
| Q12 | | Are you currently married? | Yes 1  No 0  No response -99 |  |
| **Section 2 – Reproduction, Pregnancy & Fertility Preferences** | | | | |
| Q13 | | Have you ever given birth? | Yes 1  No 0  No response -99 | 1 go to Q14  0 or -99 go to Q15 |
| Q14 | | How many times have you given birth? | \| Number \|  \| \| --- \| --- \| |  |
| **Section 3 – Contraception** | | | | |
| Q15 | Now I'd like to ask you some questions about family planning methods. I'll ask if you've heard them before. The methods are implants, injectables, the pill, condoms, IUD or IUD.  The first method: implants. Have you ever heard of the contraceptive implant?  PROBE: Women can have one or several small rods placed in their upper arm by a doctor or nurse, which can prevent pregnancy for one or more years. | | Yes 1  No 0  No response -99 |  |
| Q16 | The second method: injectables. Have you ever heard of injectables?  PROBE: Women can have an injection by a health provider that stops them from becoming pregnant for one or more months. | | Yes 1  No 0  No response -99 |  |
| Q17 | The third method: pills. Have you ever heard of the (birth control) pill?  PROBE: Women can take a pill every day to avoid becoming pregnant. | | Yes 1  No 0  No response -99 |  |
| Q18 | The fourth method: condoms. Have you ever heard of condoms?  PROBE: Men can put a rubber sheath on their penis before sexual intercourse. | | Yes 1  No 0  No response -99 |  |
| Q19 | The fifth method: IUD. Have you ever heard of the IUD?  PROBE: Women can have a loop or coil placed inside them by a doctor or a nurse. | | Yes 1  No 0  No response -99 |  |
| Q20 | Are you or your partner currently doing something or using any method to delay or avoid getting pregnant? | | Yes 1  No 2  No response 3 | 1 go to Q21  2,3 go to Q23 |
| Q21 | Which method are you using? | | Implant  3  IUD  4  Injectables 5  Pill  7  Male Condom  9  Other modern -55  No response -99 | 3-9 go to Q22  -99 go to Q27 |
| Q22 | You are using [CURRENT METHOD]. Where did you or your partner get it at that time? | | National Hospital 1  Medical centre with surgical antenna (CMA) 2  Health and Social Promotion Centre (CSPS) 3  Maternity 4  Pharmacy 5  Shop / market / supermarket 6  Other 7 | Go to Q27 |
| Q23 | In the last 12 months, have you ever done something or used a method to delay or avoid getting pregnant? | | Yes 1  No 2  No response 3 | 1 go to Q24  2, 3 go to Q25 |
| Q24 | Which method did you use most recently? | | Implant  3  IUD  4  Injectables 5  Pill  7  Male Condom  9  Other modern -55  No response -99 | All go to Q9 |
| Q25 | Have you ever done anything or tried in any way to delay or avoid getting pregnant? | | Yes 1  No 2  No response 3 | 1 go to Q26  2 go to Q9 |
| Q26 | Which method did you first use to delay or avoid getting pregnant? | | Implant  3  IUD  4  Injectables 5  Pill  7  Male Condom  9  Other modern -55  No response -99 | Go to Q9 |
| Q27 | Do you use a second method? | | Yes 1  No 2  No response 3 | Go to Q9 |
| Q9 | What is the name of the chiefdom that you live in? | | *(Drop down list)*  *List of 49 chiefdoms in appendix* | Go to Q30 |
| Q28 | You are not eligible (quota)  Thank you Madam for your time to respond, but you are not eligible to participate in this study. | | Age (15 to 19 years), urban 1  Age (15 to 19 years), rural 2  Age (20 to 49 years), urban 3  Age (20 to 49 years), rural 4 | Go to Q31 |
| Q29 | Would you like to be called back later today or tomorrow afternoon? | | Later today *(Thank you very much we will call you back later)* 1    Tomorrow or any other day  *(Thank you very much. We'll call you back tomorrow around 4:00 p.m.)* 2  Do not call back *(Thank you for your time and have a good evening!)* 3 | Go to Q31 |
| Q30 | Thank you very much, the investigation is now over!  You will receive your communication credit in the next few days. Have a good day! | | (Message) | Go to Q31 |
| Q31 | What is the result of this call? | | Completed the survey 1  Participant to be called back - Same day 2  Participant to be called back - tomorrow 3  Refusal (do not call back) 4  Hang up the phone 5  Age - Not eligible 6  Male - Not eligible 7  Quota - Not eligible 8  Call back in [language] 9 |  |

APPENDIX: List of Chiefdoms

| 1 | Banfora |
| --- | --- |
| 2 | Bati |
| 3 | Bobo-Dioulasso |
| 4 | Bogande |
| 5 | Boromo |
| 6 | Boulsa |
| 7 | Bousse |
| 8 | Dano |
| 9 | Dedougou |
| 10 | Diapaga |
| 11 | Diebougou |
| 12 | Djibo |
| 13 | Dori |
| 14 | Fada-N'Gourma |
| 15 | Gaoua |
| 16 | Gayeri |
| 17 | Gorom-Gorom |
| 18 | Gourcy |
| 19 | Houndé |
| 20 | Kaya |
| 21 | Kombissiri |
| 22 | Kongoussi |
| 23 | Koudougou |
| 24 | Koupela |
| 25 | Leo |
| 26 | Manga |
| 27 | Nouna |
| 28 | Orodara |
| 29 | Ouagadougou |
| 30 | Ouahigouya |
| 31 | Ouargaye |
| 32 | Pama |
| 33 | Po |
| 34 | Reo |
| 35 | Sapouy |
| 36 | Sebba |
| 37 | Sindou |
| 38 | Solenzo |
| 39 | Tenkodogo |
| 40 | Titao |
| 41 | Toma |
| 42 | Tougan |
| 43 | Yako |
| 44 | Ziniare |
| 45 | Zorgho |
| -99 | Ne Sais Pas |
