# Supplementary material for: Comparability of modern contraceptive use estimates between a face-to-face survey and a cellphone survey among women in Burkina Faso
Source: PLoS One. 2020 May 13;15(5):e0231819. doi: 10.1371/journal.pone.0231819 (PMC7219703; doi:10.1371/journal.pone.0231819)
Supplement: Attachment — Submitted filename: Plos ONE Reviewer comment responses_2Dec2019.docx [file pone.0231819.s002.docx]

**Reviewer #1:**
1- What are the information in first column? Are they age categories?

*Thank you for your careful attention to Table 1. Yes, this column is age groups. I have changed the column heading accordingly. Line 159*

2- Page 15, line 317, “significance using an alpha of 0.05” should be written “less than 0.05”.

*We have made this change. Line 310*

3- Page 20, line 347, please include SD or SE when you report the mean.

*We added the standard error to the text that refers to average age. Line 341*

4- Table 4, please clarify different marital status as married and non-married.

*We prefer to refer to our respondents as “in union” or “not in union” as in the setting (Burkina Faso) many people may not be married but are co-habiting, which we refer to as “in union” because they are not legally married. We will keep this classification as is, as this is also the classification the Demographic and Health Survey uses, which is our reference standard. Lines 276, 277 and Table 4.*

5- Table 5 can be deleted as its information has been explained in text completely.

*Point well taken. I have deleted Table 5.*

6- Table 6, some figured are bold which probably shows the significance but p-value is not reported.

*We have added a footnote at the bottom of the table that the bold denotes statistical significance less than 0.05. We report the confidence intervals instead of the p-value as we find the confidence interval to be more telling information than the p-value. Table 6.*

7- Discussion should show the application of the study results.

*We added a more explicit explanation of the application of study results in two areas of the discussion section: lines 411 – 414 & lines 464 – 466.*

**Reviewer #2:**

The authors present an important issue relevant for public health and epidemiological data collection. In the present article, the main aim was to assess survey errors when comparing computer assisted telephone interviews (CATI) to FTF surveys for modern contraceptive use in Burkina Faso. As the first of its kind, this paper is essential given the recent increase in cell phone ownership in low to middle income countries (LMIC). I commend their brilliant presentation and recommend this article to be considered for publication.

My only comment relating to this article is given below:

The authors clearly pointed out that the difference in contraceptive prevalence rates could not be fully explained owing in part to the small number of demographic variables available in the RDD survey. Other key indicators that influence the use of modern contraceptives in LMIC settings include the health service environment, physical infrastructure, and prevailing cultural beliefs surrounding health care seeking. Were any of these assessed as part of the study? It will be interesting to see how any of these other indicators contribute to the difference in prevalence rates between the two surveys.

*Thank you for your feedback. Unfortunately we did not collect data on key indicators you mention above. We added a sentence in the discussion section about how these variables could better explain the difference in our outcomes of interest. See lines 448-450.*

**Reviewer #3:**

I do not think the manuscript is written in a scientific manner and there is a lack of consistency across the manuscript. The comparison of both data and their interpretations are difficult to understand. Moreover, this is not a suitable manuscript for Plos One readers.

**Academic Editor:**

1) There is lack of consistent information

*This statement does not provide details about where there is perceived inconsistent information*

2) At some places the statements are not clearly presented

*This statement does not provide details about where there is perceived inconsistent information*

3) The findings and the interpretation of data don’t match

*This statement does not provide details about where there is perceived inconsistent information*

4) Variables used in the research are not clearly defined

*The AAPOR variables used (Subheading “Measure – defining call outcomes from RDD survey using AAPOR final disposition codes”) follow the same guidelines as used by L’Engle et al. published by PLOSOne in 2018. Furthermore, the outcome variables also follow the AAPOR guidelines.*

*The dependent variable, modern contraceptive use, follows the definition of modern contraceptive use given by the Demographic and Health Survey, which is the reference standard. The independent variables, which are commonly used socio-demographic variables, are defined between lines 272 – 279.*
